# Influence of Spray Angle on Scratch Resistance of Cold-Sprayed SS316L Deposits

**DOI:** 10.3390/ma18020431

**Published:** 2025-01-17

**Authors:** Avneesh Kumar, Marek Vostrak, Sarka Houdkova

**Affiliations:** Research and Testing Institute Pilsen, 30100 Plzen, Czech Republichoudkova@vzuplzen.cz (S.H.)

**Keywords:** scratch testing, cold spray, SS316L, fracture toughness

## Abstract

In this study, we investigated the effect of spray angle on the microstructure, bonding quality, and scratch resistance of cold-sprayed SS316L coatings on SS304 substrates. The coatings were deposited at spray angles of 45°, 60°, 75°, and 90° using a high-pressure cold spray system. A comprehensive analysis of the relationship between the spray angle and coating properties was conducted, with a particular focus on fracture toughness and porosity. Scratch testing, combined with real-time acoustic emission monitoring, enabled the precise identification of failure mechanisms and the assessment of coating integrity. The results indicate that microhardness and porosity are significantly influenced by the spray angle. The highest microhardness was achieved at a 45° angle, while a 90° angle resulted in the lowest porosity and superior bonding due to superior normal impact velocity. Fracture toughness was found to correlate with microstructural cohesion and particle deformation. Optimizing the incidence angle improved the coating performance by balancing strain hardening and ductility, thereby reducing the risk of premature failure. These findings are particularly relevant for industrial applications, where wear resistance and high-quality bonding are critical, such as in aerospace, automotive, and marine sectors. By adjusting the spray angles, manufacturers can enhance the longevity and reliability of the coated components, thus reducing maintenance costs and improving performance. This research highlights the importance of process parameters in achieving durable, high-quality coatings and emphasizes scratch testing as an effective, sustainable, and semi-destructive evaluation method for coating integrity.

## 1. Introduction

Cold spray coatings offer immense potential for industrial applications due to their ability to produce dense, durable layers without melting the base material. It enables solid-state deposition of micron-sized particles in a layer-by-layer manner. While seemingly straightforward, this process involves complex interactions at both the particle–substrate and particle–particle interfaces. The bonding mechanisms in cold spray have been thoroughly investigated in prior studies [1,2,3,4,5,6,7,8], providing a solid basis for analyzing the cold spray coating properties. This study focuses on cold-sprayed SS316L coatings, which are increasingly utilized in demanding sectors, such as marine, nuclear, and aerospace, due to their excellent corrosion resistance and mechanical properties.

Previous research has demonstrated the successful deposition of SS316L coatings using cold spray, achieving favorable microstructural and mechanical properties. For example, Spencer et al. [9] deposited SS316L onto AZ91E magnesium alloy, optimizing its microstructure for corrosion resistance by employing a mixed particle size distribution, although the deposition efficiency remained below 30%. In contrast, Villa et al. [10] achieved a 90% deposition efficiency by optimizing the gas pressure and temperature, resulting in a hard and wear-resistant coating tailored for wear applications. Previous research also highlights the influence of cold spray parameters on coating quality, yet the optimization of spray angles to improve critical properties, such as fracture toughness, microhardness, and porosity, remains underexplored.

The particle spray (impact) angle is a decisive factor influencing coating performance by altering the particle deformation, bonding quality, and microstructure. As the spray angle changes, the balance between the normal and tangential impact velocities significantly affects the intersplat cohesion and related coatings performance. Among the many properties critical to coating performance, scratch resistance plays a pivotal role in determining their structural integrity and long-term durability.

The scratch test is a semi-destructive method widely employed to assess material resistance to surface damage by scratching [11]. In thermal spray coatings, this technique is particularly useful for assessing adhesion strength [12,13]. During the test, an indenter moves across the coated surface under either a constant or gradually increasing load. Failure, typically in the form of delamination or cracking, occurs at a specific load known as the critical load [14], which serves as an important indicator of bonding strength. These failures often result from internal stresses exceeding the bond strength at the coating–substrate interface due to elastic deformation. Despite being semi-destructive, the scratch test is highly effective for evaluating the structural integrity and bonding quality of coatings in various applications [15]. However, most research has concentrated on hard and brittle coatings, leaving limited and often conflicting data for ductile coatings and substrates [16,17,18]. The scratch performance of cold-sprayed coatings is intrinsically linked to their microstructure and mechanical integrity. Critical properties such as hardness, porosity, and intersplat bonding play a decisive role in determining the scratch resistance and overall durability under working conditions. In cold spray coatings, the splat interfaces are crucial, as the balance between plastic deformation and interfacial bonding largely governs the scratch resistance. Fine-tuning these interfaces through precise control of process parameters not only enhances mechanical strength but also improves the capacity of the coatings to endure the complex stress states encountered during scratch testing. A thorough understanding of the process parameters and their effects is essential for achieving high-quality repairs, coatings, and three-dimensional components. This is especially true in cold spray technology, where the scratch response of coatings plays a vital role in meeting the demands of advanced engineering applications.

One of the key optimization strategies involves assessing fracture toughness, a critical property that reflects the ability of a material to absorb energy before failure during scratch loading. Fracture toughness significantly influences the resistance of a coating to deformation, cracking, and delamination, which is crucial for ensuring long-term durability. In cold-sprayed coatings, the particle spray angle has a profound effect on toughness and durability by shaping intersplat bonding and overall structural integrity [19,20]. This parameter is particularly important in repair applications, where mechanical performance under stress is a primary concern. SS316L is a popular choice for repair due to its excellent formability, toughness, and weldability [21]. While considerable research has focused on optimizing the cold spray parameters to enhance the corrosion and wear resistance of SS316L coatings [22,23,24], there is a noticeable gap in studies addressing the optimization of spray angles to improve toughness and durability. Filling this gap is critical for expanding the potential of cold spray technology for use in demanding engineering applications.

For evaluating fracture toughness through the scratch method, Akono et al. [25,26] introduced a linear elastic fracture mechanics (LEFM) model, tested across various materials, including polymers, metals, and ceramics. This model is based on dimensional analysis principles. Akono’s model has been a well-established approach since its introduction in 2011. It provides a reliable starting point for evaluating the fracture toughness. This model was chosen because it has been widely used by researchers for material testing. However, Akono’s model does not consider the effect of transition depth while calculating fracture toughness, which is crucial for metallic materials. More recently, Zhang et al. [27] proposed a new approach that integrates the size effect law with the dimensional model, demonstrating its applicability to metallic materials with high fracture toughness. In this study, both models are applied to assess the fracture toughness of cold-sprayed coatings, and their results are compared and discussed.

The primary objective of this work is to optimize cold-sprayed SS316L coatings by performing micro-scratch testing at different particle spray angles. To the best of the authors’ knowledge, no prior studies have explored the effect of spray angle on the fracture toughness of SS316L coatings on SS304 substrates, as evaluated through micro-scratch testing. This study aims to address this gap and provide valuable insights into the role of spray angle in enhancing coating performance.

## 2. Materials and Methods

**Figure 1:**
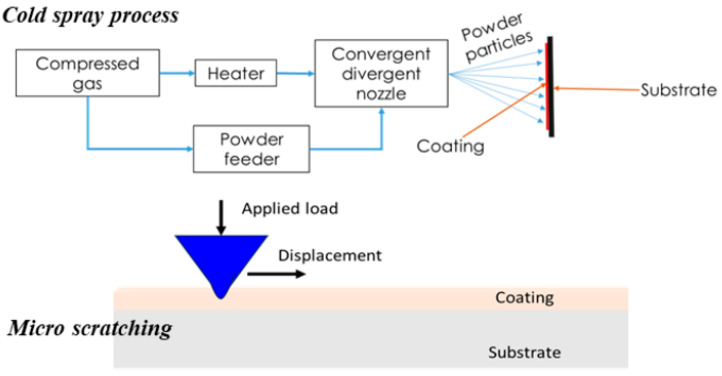
Schematic representation of cold spray process and scratch testing method.

The coatings were deposited on SS304 steel substrates with dimensions of 100 mm × 100 mm × 5 mm. Prior to deposition, the substrates were subjected to grit blasting and then cleaned with acetone to ensure optimal surface preparation. The SS316L powder used for coating deposition was sourced from Oerlikon Metco, Pfaeffikon, Switzerland, featuring a spherical shape with a particle size range of 15 to 45 µm. A high-pressure cold spray system (6/11 EvoCSII, Impact Innovations GmbH, Rattenkirchen, Germany) was employed for deposition, using nitrogen as the processing gas. Schematic representation of the cold spray process is shown in Figure 1. While parameters such as gas pressure, temperature, powder feed rate, and standoff distance were kept constant, the spray angle was systematically varied from 45° to 90°, as detailed in Table 1.

Once the coatings were deposited, they were sectioned, cold-mounted, and polished to prepare for analysis. The microstructure of the coatings was then examined using scanning electron microscopy (SEM, JEOL, JSM-6490LV, Tokyo, Japan). To assess porosity, high-magnification optical images were captured and analyzed with ImageJ 1.48v software, with five images analyzed per sample to ensure consistency. Furthermore, microhardness testing was conducted on the polished cross-sections using a Vickers indenter, where ten indentations were made for each sample at a load of 300 g, and each indent was held for 10 s to ensure accurate measurements.

Before conducting scratch testing, the samples were polished to remove any influence from coating roughness. The scratch tests were carried out using a micro scratch tester (Universal Tribometer Mod. UMT-2-1, Bruker, Billerica, MA, USA), which was equipped with a Rockwell diamond indenter with a 200 µm tip radius. A schematic representation of scratching method is shown in Figure 1. A progressively increasing load, reaching up to 100 N, was applied over a 5 mm scratch length on the coating surface. Each coating underwent three scratch tests. Each test consisted of three distinct phases: an initial pre-scan at 2 N, the primary scratch pass, and a post-scan. During the testing, key parameters such as frictional force, penetration depth, coefficient of friction (CoF), and acoustic emission were continuously monitored. These data were later analyzed to determine the critical load at failure and to assess the fracture toughness of the coatings. The fracture toughness of the resulting coatings was calculated using two distinct models: one proposed by Akono et al. [25] and the other by Zhang et al. [27].

Akono et al. [25]:

(1) $$K_{c}=\;\frac{F_{x}}{\sqrt{2pA}}$$

where *F_x_* is the frictional force, *p* is the perimeter of the indenter, and *A* is the horizontal load projected contact area of the indenter.

(2) p=πdtan⍵

where *d* and ⍵ are the penetration depth and half apex angle of the indenter, respectively.

(3) $$A=R^{2}{Cos}^{-1}\left\{1-\frac{d}{R}\right\}-\;\left(R-d\right)\;\sqrt{2Rd-d^{2}}$$

where *R* is the radius of the Rockwell indenter.

Zhang et al. [27]:

(4) $$\frac{1}{K_{c}^{2}}=\;\frac{2pA}{F_{x}^{2}}-\;\frac{2p}{A(B^{’}U_{T}{)}^{2}}$$

(5) $$B^{\prime }U_{T}=\;\frac{F_{x}}{A}\;\times \;{\left(1+\frac{A}{2pl_{o}}\right)\;}^{0.5}$$

where *l_o_* is the transition depth of the indenter.

## 3. Results and Discussion

**Figure 2:**
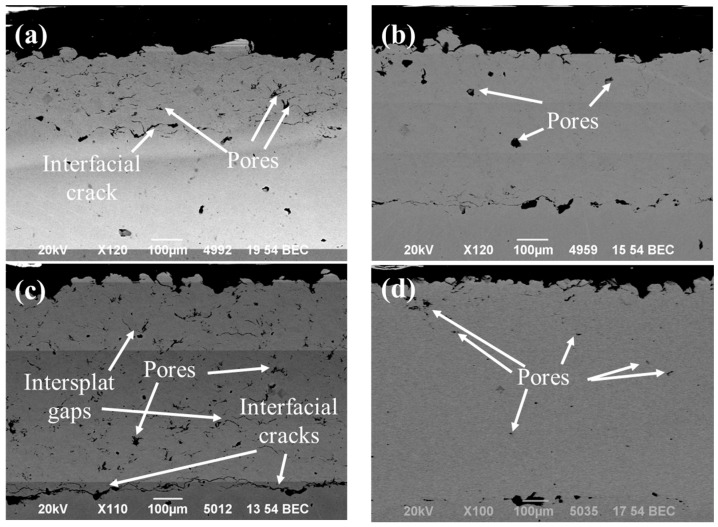
High-magnification scanning electron microscopic images at different spray angles of robotic arm to the substrate: (**a**) 45°, (**b**) 60°, (**c**) 75°, and (**d**) 90°.

Figure 2 presents the cross-sectional micrographs of SS316L cold spray coatings applied to SS304 substrates, highlighting the influence of varying deposition angles (45° to 90°) on the microstructure. The analysis of the images reveals significant differences in microstructure, coating–substrate interface quality, and coating thickness, emphasizing the importance of deposition parameters. At a 45° spray angle, the microstructure displayed intersplat gaps and voids along the coating–substrate interface. Notably, this angle produced the smallest coating thickness among all samples (Table 2), despite the same number of spray layers. These results suggest insufficient particle deformation and energy transfer, leading to weak bonding. The coatings deposited at a 60° spray angle demonstrated noticeable improvement, with a well-bonded microstructure and intersplat gaps largely restricted to the coating–substrate interface. The increased coating thickness observed at this angle indicates better particle deformation and adhesion. At 75°, the coatings exhibited mixed characteristics. While fewer intersplat gaps were observed compared to the 45° angle, they were still more prevalent than in the 60° sample. Additionally, interfacial cracks were present, indicating poor adhesion to the substrate. Despite these issues, the coating thickness exceeded that of the 45° and 60° samples, suggesting improved particle deformation and retention at this angle. The most favorable results were obtained at a 90° spray angle, where the coatings demonstrated a uniform microstructure with minimal intersplat gaps and a defect-free interface. This angle also produced the thickest coating, reflecting efficient particle deformation and retention. Similar findings were reported by Singh et al. [28] for the IN718 coatings produced via cold spray, further validating the significance of spray angle in optimizing the coating performance.

The variations in coating microstructure and adhesion with changing spray angles highlight the intricate relationship between particle dynamics and cold spray deposition efficiency. At oblique spray angles, the particle velocity decomposes into two components: a normal velocity perpendicular to the substrate and a tangential velocity parallel to it, as detailed by Seng et al. [29] in their study on Inconel 718 coatings. As the spray angle decreases, the normal velocity component diminishes, while the tangential component increases, influencing particle deformation within the coating. At a 45° spray angle, the tangential velocity is likely at its peak, while the normal velocity is at its lowest among the cases studied. This results in minimal particle deformation or unidirectional deformation (sliding of particle), leading to limited metallurgical bonding and mechanical interlocking between splats. Although the microstructure may show significant deformation, this is primarily attributed to tamping and hammering effects from subsequent layers. The poor bonding observed at this angle can be attributed to the high tangential or sliding impact velocity of particles, leading to reduced deposition efficiency and thinner coatings compared to other angles. At 60°, the microstructure improves, and the coating thickness increases, suggesting a better balance between the normal and tangential velocities. This balance enhances particle deformation and bonding. The behavior observed at 75° represents a transitional zone where increased normal impact velocity and decreased tangential impact velocity result in thicker coatings but also introduce structural defects, such as intersplat gaps and interfacial cracks. These defects likely originated from the high residual stresses associated with greater coating thickness and tangential impact velocity, highlighting the importance of precise control over impact dynamics to minimize such issues. At 90°, the normal impact velocity reaches its maximum, and the tangential component approaches zero, leading to optimal particle deformation and adhesion. Despite the potential for elevated residual stresses at this angle, the resulting strong bonding and deformation balance contribute to superior coating quality. This is supported by minimal intersplat gaps, excellent interfacial bonding, and the highest coating thickness among all the samples. These findings emphasize the advantages of normal impacts in producing dense, adherent coatings with improved mechanical integrity and deposition efficiency.

**Figure 3:**
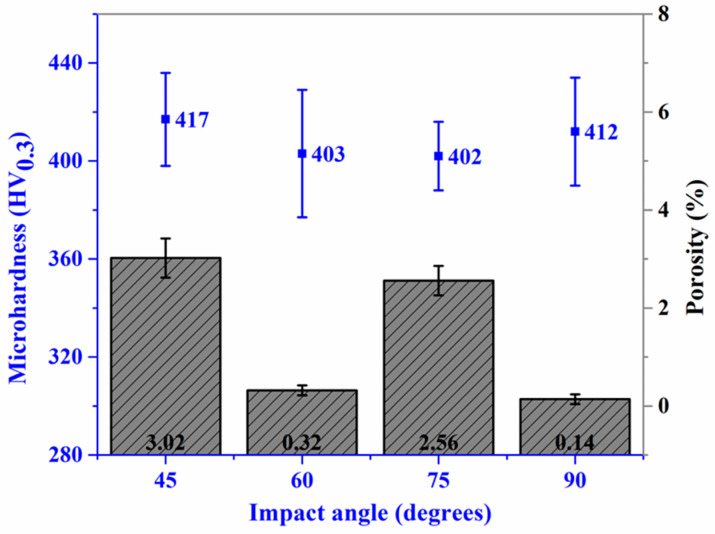
Microhardness and porosity measurements for cold-sprayed SS316L coatings on SS304 substrate at a varying spray angle of the robot to the substrate.

Figure 3 illustrates the microhardness and porosity results of the studied coatings. The data reveal that the highest average microhardness (417 HV) is achieved at a 45° spray angle. A 3.35% decrease in microhardness is observed as the spray angle increases from 45° to 60°, after which it remains relatively stable between 60° and 75°. Notably, the microhardness rises by 3% when the spray angle increases from 75° to 90°. The coatings deposited at 60° and 90° exhibit the lowest porosity, with values that are nearly identical. Additionally, these coatings show a uniform microstructure, contributing to improved splat bonding and reduced porosity. In contrast, the coatings deposited at 45° and 75° demonstrate the highest and comparable porosity levels. The 45° coating exhibits greater porosity variation, indicative of a heterogeneous microstructure and insufficient plastic deformation of splats.

In the above discussion, it was noted that as the spray angle of the spray gun decreases relative to the substrate, the normal impact velocity decreases while the tangential impact velocity increases. These changes significantly affect particle deformation upon impact and, consequently, intersplat bonding. The coating deposited at the lowest spray angle (45°) exhibited the highest microhardness and porosity. This outcome is attributed to severe plastic deformation driven by the hammering effect, which increases the dislocation density and strain hardening within individual splats, leading to elevated microhardness. However, despite the high microhardness, the poor intersplat bonding quality arises from insufficient particle deformation during the initial pass, resulting in gaps and defects at splat interfaces. The high tangential velocity restricted metallurgical bonding and mechanical interlocking between splats due to sliding between splats [29]. At a spray angle of 60°, particle deformation increased compared to the 45° case, primarily due to the relatively higher normal impact velocity and reduced tangential impact velocity. This led to a uniform microstructure with improved intersplat bonding. However, the greater coating thickness per pass reduced the hammering effect, resulting in lower microhardness. Further increasing the spray angle to 75° introduced higher residual stresses due to increased coating thickness per pass [30]. Combined with the effects of tangential impact velocity, this resulted in poor intersplat bonding and a porous microstructure, with hardness comparable to the previous case. At a normal spray angle (90°), the normal impact velocity was maximized, promoting optimal particle deformation and excellent intersplat bonding. Consequently, a dense microstructure with high microhardness was achieved. Although the thicker coating layers increased the residual stresses, these were mitigated by the strong intersplat bonding.

### 3.1. Scratch Testing on the Coating Surface

#### 3.1.1. Frictional Force and Penetration Depth During Progressive Loading

**Figure 4:**
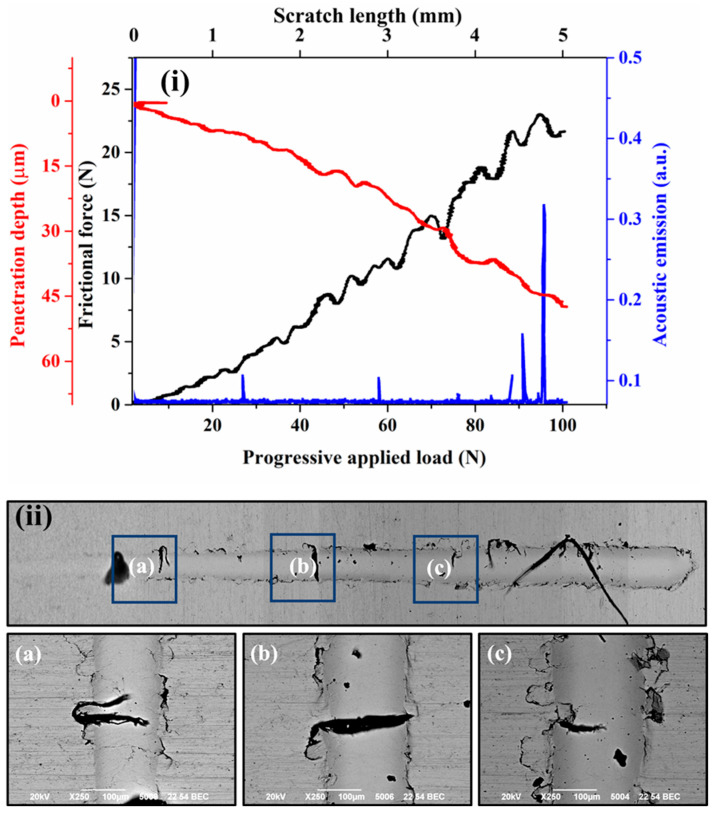
Scratch surface response of the cold-sprayed SS316L coating on SS304 substrate produced at spray angle of 45° (**i**) Frictional force and penetration depth response (**ii**) SEM images of the scratch (**a**)–(**c**) High magnification SEM images at different locations.

**Figure 5:**
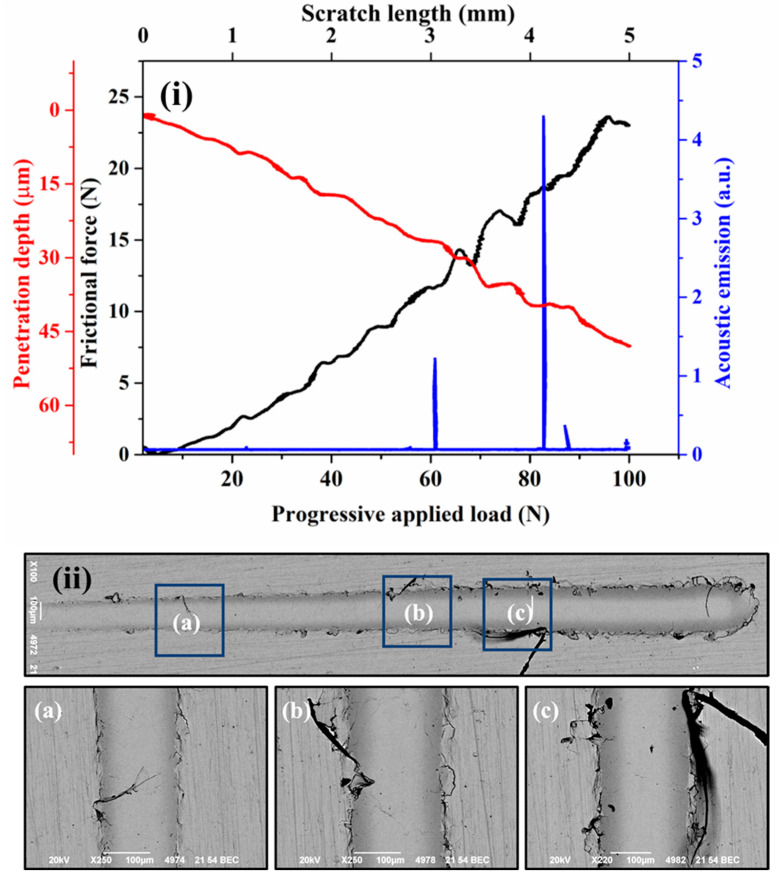
Scratch surface response of the cold-sprayed SS316L coating on SS304 substrate produced at spray angle of 60° (**i**) Frictional force and penetration depth response (**ii**) SEM images of the scratch (**a**)–(**c**) High magnification SEM images at different locations.

**Figure 6:**
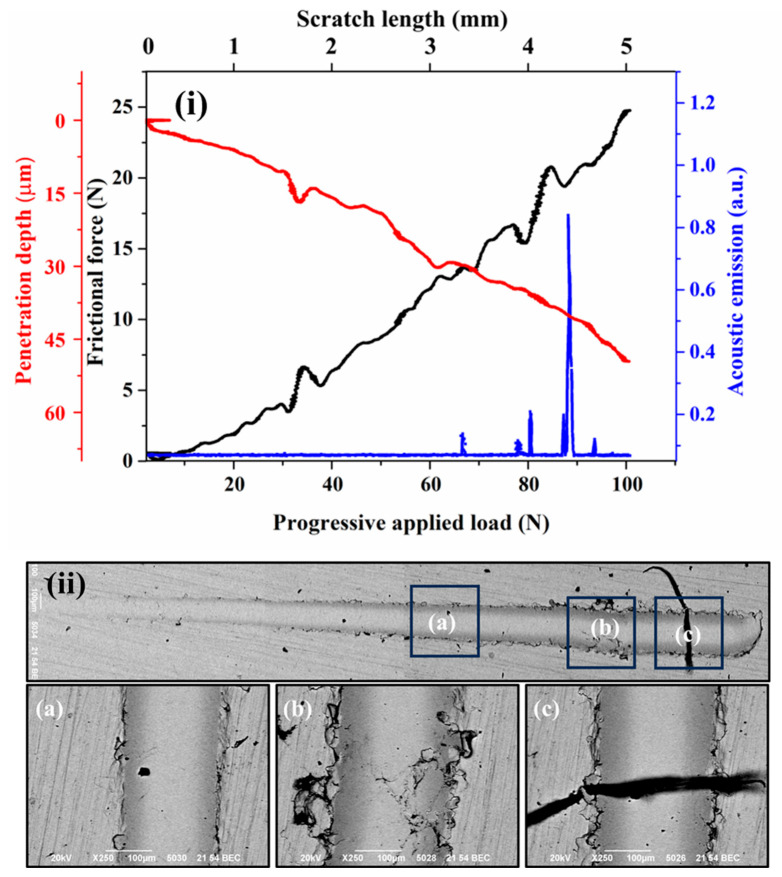
Scratch surface response of the cold-sprayed SS316L coating on SS304 substrate produced at spray angle of 75° (**i**) Frictional force and penetration depth response (**ii**) SEM images of the scratch (**a**)–(**c**) High magnification SEM images at different locations.

**Figure 7:**
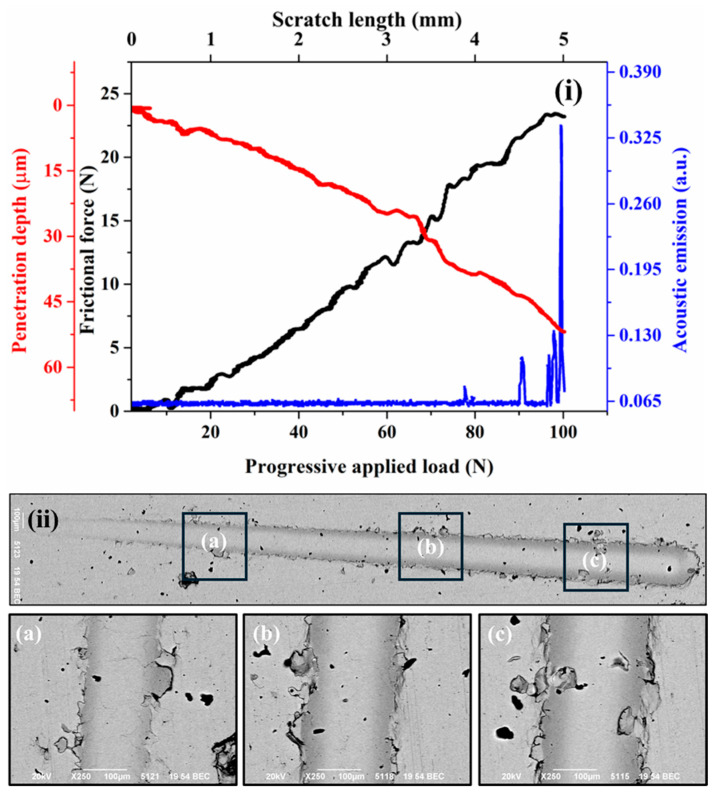
Scratch surface response of the cold-sprayed SS316L coating on SS304 substrate produced at spray angle of 90° (**i**) Frictional force and penetration depth response (**ii**) SEM images of the scratch (**a**)–(**c**) High magnification SEM images at different locations.

Figure 4i, Figure 5i, Figure 6i, and Figure 7i present the frictional force, penetration depth, and acoustic emission signals recorded during progressive scratch tests on coatings deposited at spray angles of 45°, 60°, 75°, and 90°, respectively. The corresponding SEM images of the scratch tracks are shown in Figure 4ii, Figure 5ii, Figure 6ii, and Figure 7ii. The results indicate minor fluctuations in frictional force and penetration depth up to the applied loads of 27 N, 62 N, 67 N, and 78 N for the respective spray angles. These initial fluctuations suggest resistance occurring from the inter-splat boundaries or plastic deformation of splats under the applied load. At lower loads, the deformation primarily occurs through compaction of the surrounding material and porosity reduction. Beyond these load thresholds, the scratching process enters a second phase, characterized by more pronounced fluctuations in frictional force and penetration depth, accompanied by significant acoustic emission signals. These variations indicate splat debonding or the initiation and propagation of microcracks, as evidenced in the SEM micrographs. The maximum frictional force recorded across all coatings remains nearly the same for all the cases. Similarly, the maximum penetration depth at the highest applied load remains almost equal for all the cases.

Although the penetration depth typically correlates with surface hardness, in cold-sprayed coatings, it also reflects components such as cohesion quality and the presence of splat boundaries. Thus, the measured penetration depths provide insights into both the hardness and structural integrity of the coatings. All coatings exhibit cracking or debonding around the scratch tracks under the applied loads, except for the coating deposited at a 90° spray angle. This superior performance is attributed to its optimized microstructure, resulting from optimal particle deformation due to the highest normal impact velocity and minimal tangential impact velocity. The acoustic emission signals were used to identify the transitions in penetration depth during scratch testing, which directly influenced the calculation of fracture toughness using the Zhang et al. [27] approach.

**Figure 8:**
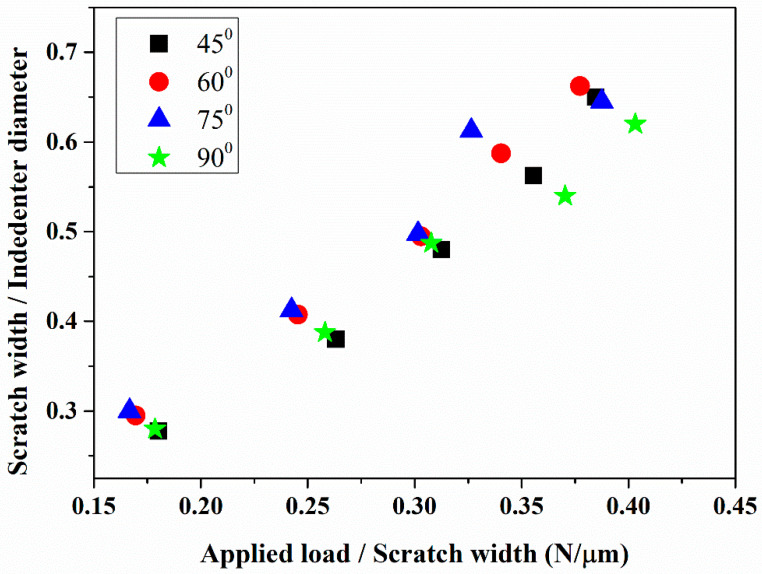
Relationship between scratch width ratio and the ratio of applied load to the scratch width for different spray angles.

Figure 8 demonstrates the correlation between the scratch width to indenter diameter ratio (W/D) and the applied load to scratch width ratio (P/W). The relevant data are reported in Table 3. The mechanical response and microstructural integrity of cold-sprayed SS 316L coatings were evaluated based on scratch test behavior and the onset of cracking. The coating applied at a 45° angle exhibited the lowest W/D ratio and the highest P/W ratio up to an applied load of 60 N. Beyond this load, from 60 N to 80 N, the coating’s behavior shifted. A notable observation was the presence of a thick intersplat crack at 25 N. Similarly, the coating deposited at 60° followed the same trend up to 60 N; however, its performance diverged afterward. Across all loads, this coating showed a higher W/D ratio and a lower P/W ratio compared to the 45° coating, indicating greater deformation and reduced load-bearing capacity. Cracks were first observed at 40 N in this sample. Contrary to this, the coating deposited at a 75° angle had the lowest P/W ratio and the highest W/D ratio, closely matching the behavior of the coating deposited at 60° up to 60 N. At 80 N, the W/D ratio increased significantly, while the P/W ratio dropped sharply. By 100 N, this coating displayed the second-lowest W/D and P/W ratios, indicating a relative improvement in the load-bearing capacity and deformation resistance beyond 80 N. A thick crack appeared at 57 N in this coating. The results suggest that the coating deposited at 45° offers superior load-bearing capacity and resistance to deformation compared to the coatings deposited at 60° and 75°, particularly at lower loads. The 75° coating, however, demonstrates better performance at higher loads.

The coating deposited at a 90° spray angle demonstrates the second-lowest W/D ratio and the second-highest P/W ratio, closely matching the performance of the coating deposited at a 45° spray angle up to an applied load of 60 N. Beyond this load, it shows the highest P/W ratio and the lowest W/D ratio among all the investigated coatings. This indicates that the coating deposited at 90° offers comparable load-bearing capacity and deformation resistance to the coating deposited at a 45° spray angle at low to moderate loads. However, at higher loads, the coating deposited at 90° outperforms all others in both the load-bearing capacity and deformation resistance. Notably, no signs of cracking were observed in the coating deposited at a spray angle of 90° throughout the applied load range.

These differences emphasize the influence of cold spray process parameters on the coating microstructure. The superior load-bearing capacity and deformation resistance of the coatings deposited at 45° and 90° can be attributed to their higher microhardness. The cracking observed in the 45° coating is likely due to its brittleness, resulting from extensive plastic deformation caused by repeated hammering during deposition. In contrast, the absence of cracking in the 90° coating is due to favorable plastic deformation and minimal hammering effects, which are a result of greater coating thickness per pass. The inferior performance of the 60° coating can be linked to its lower microhardness and inherent brittleness, which are also a result of particle hammering. Similarly, the poorest performance was observed in the 75° coating, which can be attributed to insufficient plastic deformation, poor intersplat bonding, and unbalanced residual stresses, resulting from greater coating thickness per pass.

#### 3.1.2. Fracture Toughness Determination Using the Micro-Scratching Method

The fracture toughness of cold-sprayed coatings reflects both their deformation capacity and cohesive strength. In this study, it was evaluated using a linear elastic fracture mechanics (LEFM) approach, similar to the method proposed by Akono et al. [25], which correlates frictional force with penetration depth across various materials. This relationship, detailed in Equation (1), was employed to determine the fracture toughness. Additionally, Zhang et al. [27] introduced an updated model that accounts for transition depth in metals and alloys, as outlined in Equation (4). Both models were applied in this study to assess the fracture toughness. Before conducting these evaluations, a correlation between frictional force and penetration depth was established to analyze the frictional behavior, which is assumed to follow the model described in Equation (6).

(6) $$F_{x}=2p(d)A(d)$$

**Figure 9:**
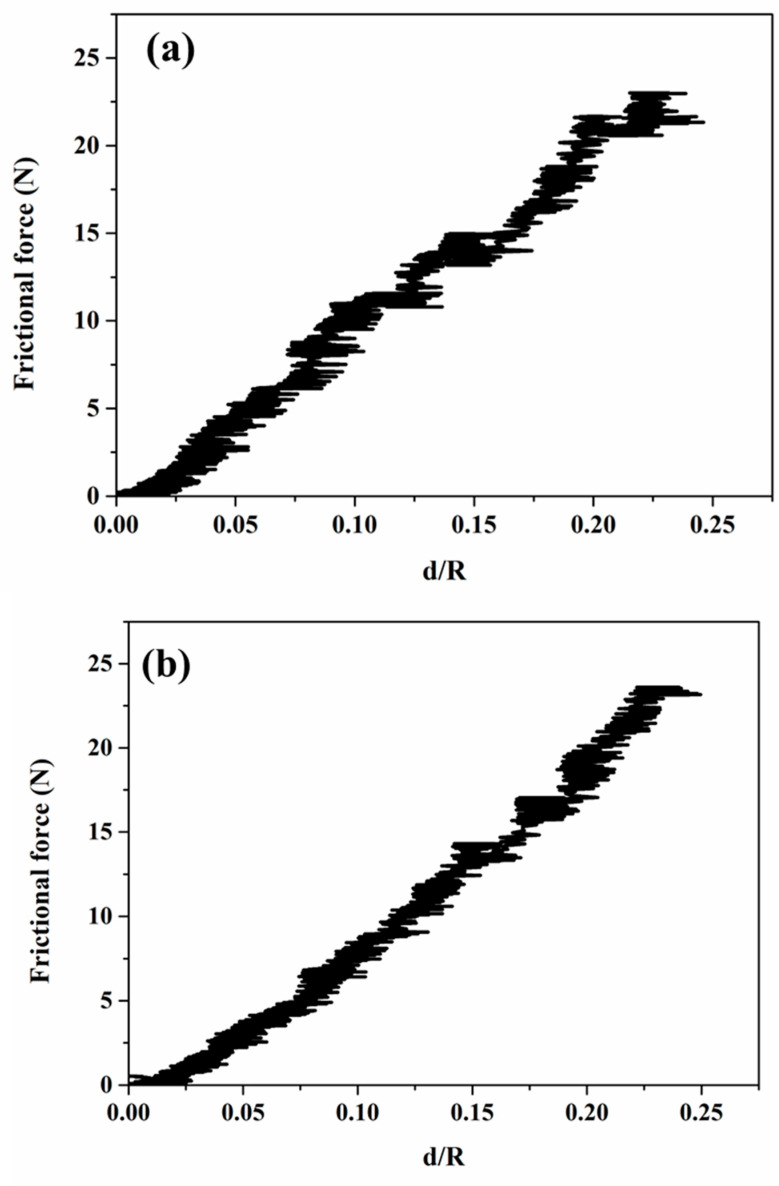

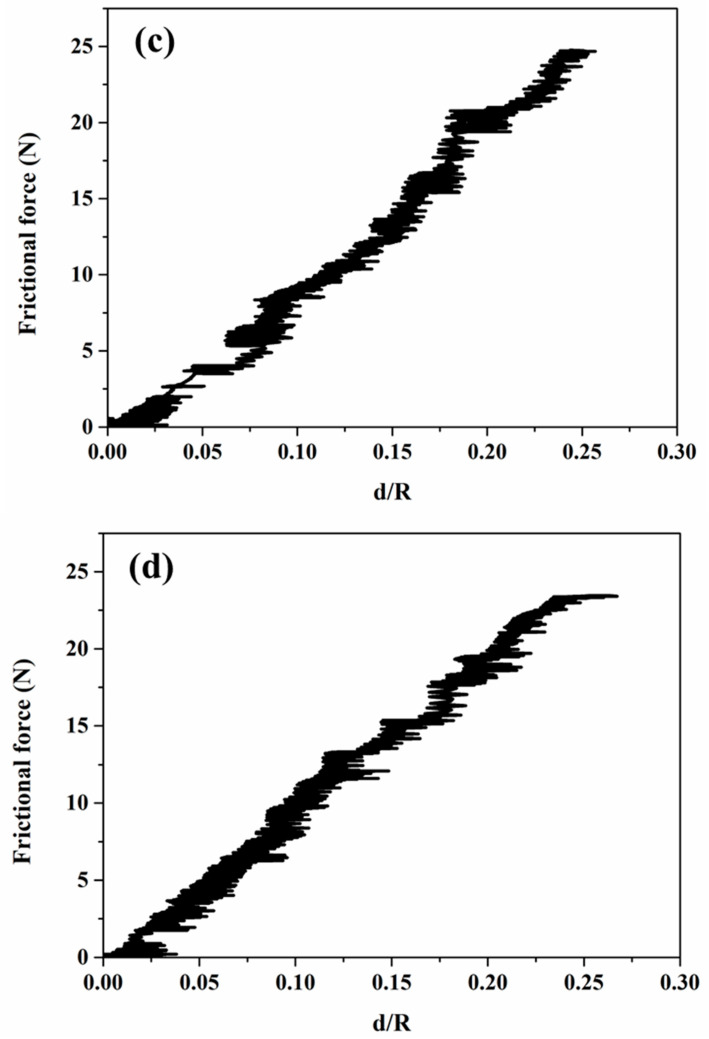
Frictional force versus penetration depth curve of cold-sprayed SS316L coatings on SS304 substrates at different spray angles of robotic arm to the substrate: (**a**) 45°, (**b**) 60°, (**c**) 75°, and (**d**) 90°.

The frictional force is considered to depend on both the perimeter of the indenter and its projected area. These values are determined using Equations (2) and (3). By simplifying Equation (6) for a Rockwell conical indenter, the resulting relationship is expressed in Equation (7). Therefore, the frictional force against the d/R ratio is plotted to understand their relationship. Figure 9 displays the relationship between the frictional force and penetration depth. The data are found to follow Equation (8) for all the cases.

(7) $$F_{x}\propto \;d^{3/2}$$

(8) $$F_{x}=a{\left(\frac{d}{R}\right)}^{b}$$

where *a* and *b* are constants.

(9) $$a=\frac{4Sin⍵}{{\left(Cos⍵\right)}^{2}}$$

For a conical indenter with a 60° angle, the theoretical value of the parameter “a” is calculated as 13.86 based on Equation (9) [26]. In this study, the parameter “b” is approximately 1.5, indicating that the conical section of the indenter primarily governs the scratching behavior. A parameter “b” closer to 1 would suggest a greater influence of the spherical portion of the indenter. This distinction sprays both the indenter’s projected area and the correlation between frictional force and penetration depth.

**Figure 10:**
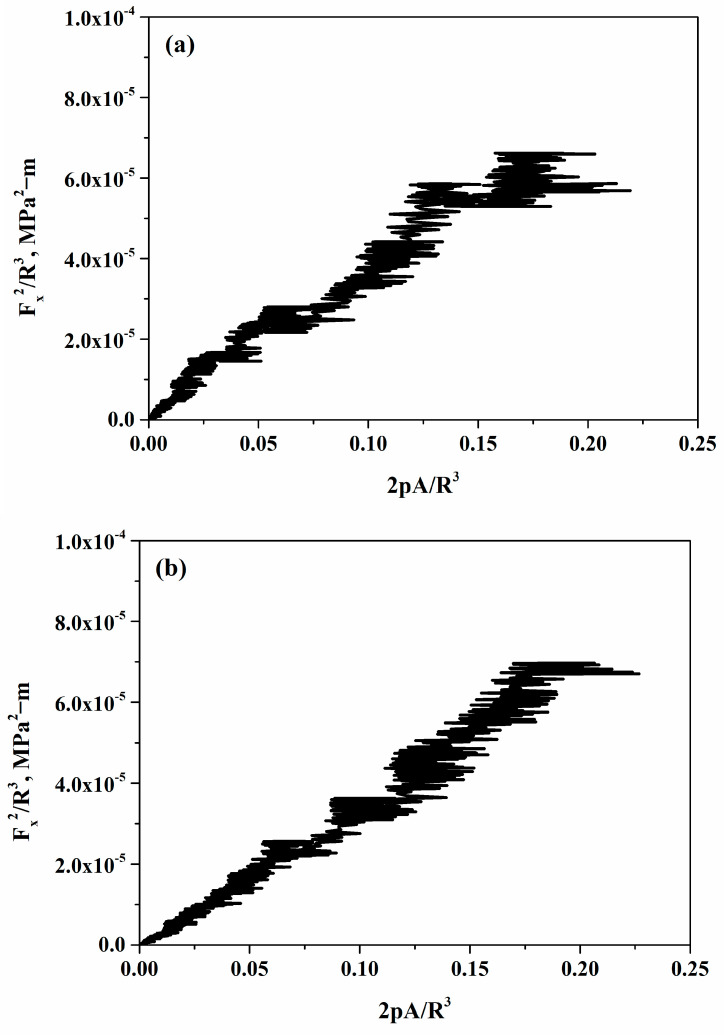

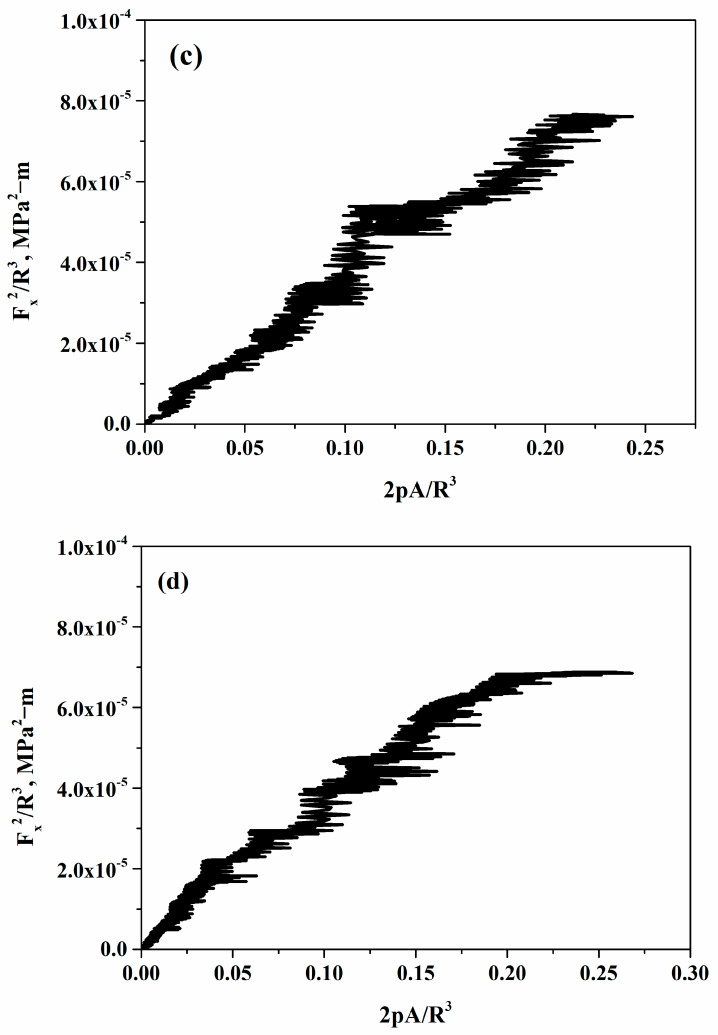
Fracture scaling of cold-sprayed SS316L coatings on SS304 substrates at different spray angles of robotic arm to the substrate: (**a**) 45°, (**b**) 60°, (**c**) 75°, and (**d**) 90°.

To further explore the influence of frictional force on the shape of the indenter, a relationship between the frictional force, the perimeter, and the horizontal projected area of the indenter was derived using dimensional analysis. The data from scratch tests were plotted (see Figure 10), and it was found to adhere to the relationship described by Equation (10), which represents a modified form of Equation (1).

(10) $$\frac{F_{x}^{2}}{R^{3}}=k_{c}^{2}\frac{2pA}{R^{3}}\;$$

Figure 10 illustrates a linear relationship between *F_x_*^2^*/R*^3^ and *2pA/R*^3^ for all the cases. This linearity validates the use of this data for calculating the fracture toughness of cold-sprayed SS316L coatings.

**Figure 11:**
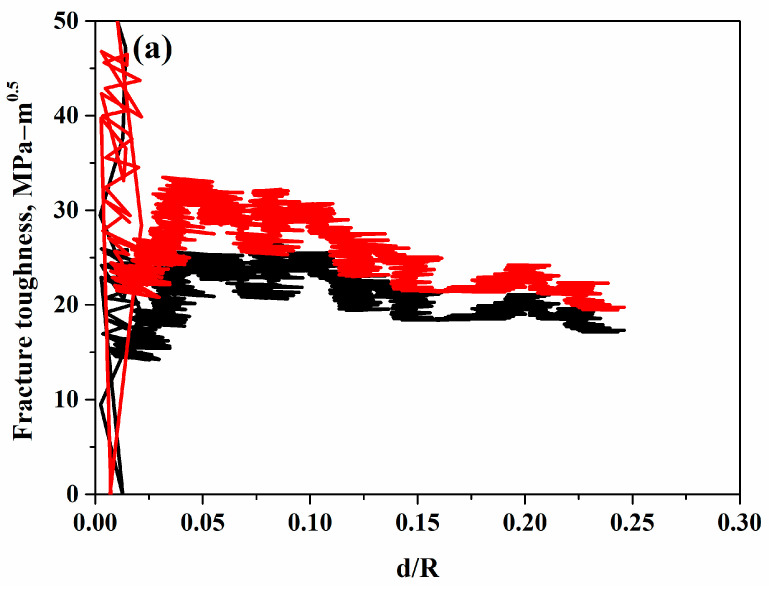

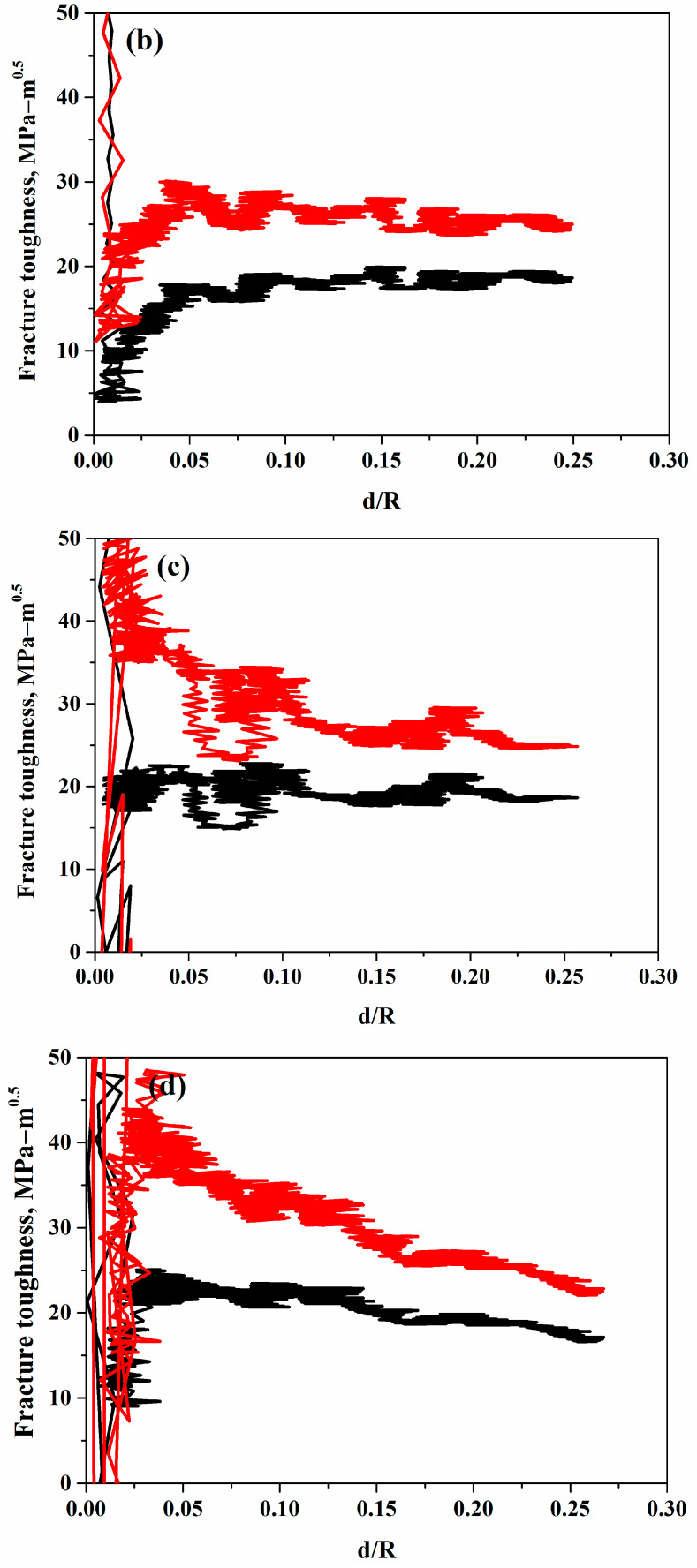
Fracture toughness versus penetration depth curves of cold-sprayed SS316L coatings on SS304 substrates at different spray angles of robotic arm to the substrate: (**a**) 45°, (**b**) 60°, (**c**) 75°, and (**d**) 90°. The red curve follows Zhang et al. [27]’s model, and the black curve follows Akono et al. [25]’s model.

**Figure 12:**
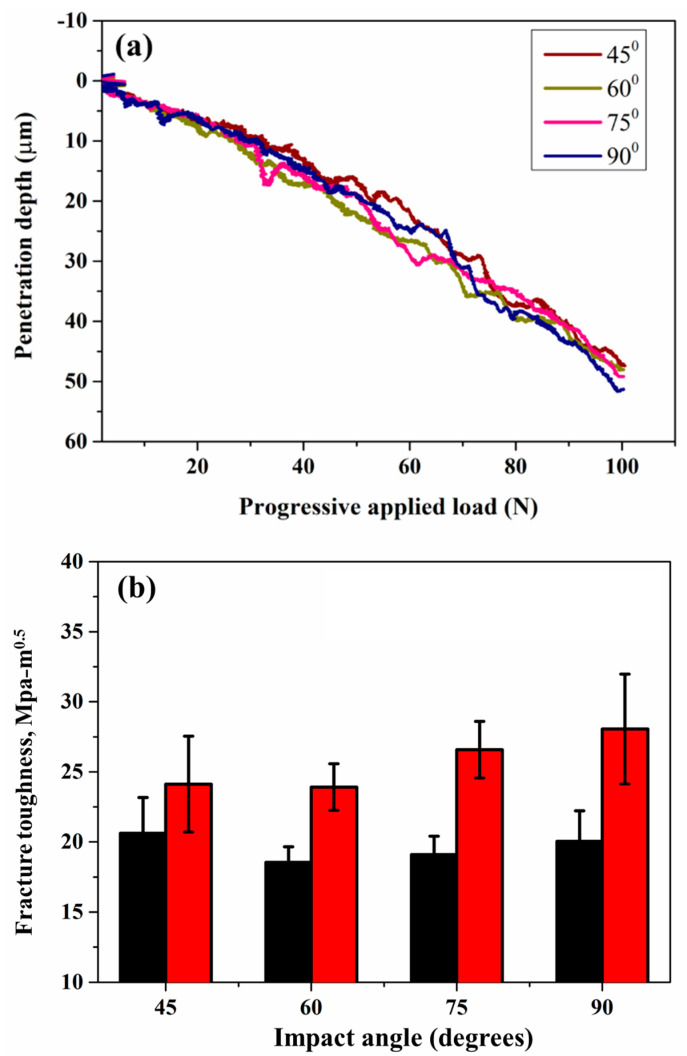
(**a**) Penetration depth versus progressively applied load curves at different spray angles of robotic arm to the substrate (**b**) Fracture toughness value for both the models at different spray angles of robotic arm to the substrate. The red represents Zhang et al. [27]’s model, and the black represents Akono et al. [25]’s model

Fracture toughness values, calculated using the methods proposed by Akono et al. [25] and Zhang et al. [27], are plotted against the normalized penetration depth (d/R) in Figure 11. The data reveal that fracture toughness consistently stabilizes, though fluctuations are observed during convergence. These fluctuations are attributed to the heterogeneous microstructure of the coatings. The observed convergence supports the reliability of Equations (1) and (3) in predicting the fracture toughness of cold-sprayed SS316L coatings. The final fracture toughness values were obtained by averaging the results from Equations (1) and (4) for penetration depths greater than half of the maximum depth, as shown in Figure 12b. Among the investigated coatings, the one deposited at a 90° spray angle exhibited the highest fracture toughness following Zhang’s model, whereas the coating deposited at a 45° spray angle offered the highest fracture toughness for Akono’s model. The coatings deposited at 60° and 75° displayed the lowest and almost equal fracture toughness following Akono’s model. The penetration depth at different spray angles under scratch testing is shown in Figure 12a.

The difference in fracture toughness values and trends between the two models highlights the role of transition depth and coating integrity in the predicted results. This influence is directly linked to crack initiation and propagation during scratch testing. As previously discussed, the fracture toughness of cold-sprayed coatings is influenced not only by surface microhardness but also by intersplat cohesion. Akono’s model corresponds with the trend observed in microhardness data, as noted earlier. In contrast, Zhang’s model considers the cohesion strength of the coating by accounting for crack initiation through transition depth. For the cold spray coatings, Zhang’s model is considered more relevant and reliable as it accounts for the effect of transition depth, which significantly depends on intersplat bonding in cold spray coatings. This characteristic is particularly crucial for ductile coatings like SS316L. Transition depth significantly influences fracture behavior as it marks the point where the deformation mechanism shifts from elastic to plastic, impacting the resistance of coatings to fracture. In the context of different spray angles, the transition depth varies due to changes in particle impact dynamics, which affect the degree of particle deformation and inter-splat bonding. At lower angles, the increased tangential component leads to shallower transition depths, reducing fracture toughness. Conversely, at higher angles, particularly 90°, the normal impact maximizes particle embedding, resulting in a deeper transition depth and enhanced fracture resistance.

## 4. Conclusions

This study investigates the quality of cold-sprayed SS316L coatings applied to SS304 substrates, emphasizing the effect of particle impact angle on coating properties. Coatings were deposited at spray angles of 45°, 60°, 75°, and 90° using a high-pressure cold spray system. A comprehensive analysis was performed to explore the relationships between process parameters, microstructure, and coating performance. Scratch testing was utilized to assess fracture toughness and bonding quality. The main findings are summarized below:

- Microhardness Dependence on Spray Angle: The spray angle was found to significantly affect the microhardness. The coating deposited at a 45° angle exhibited the highest microhardness, which progressively decreased as the spray angle increased to 75°. However, a slight increase in microhardness was observed at a 90° spray angle due to the higher normal impact velocity.
- Porosity Trends: The lowest porosity was observed at a normal spray angle of 90°. Porosity increased at a spray angle of 75° due to unbalanced residual stresses resulting from higher thickness per pass and the tangential impact velocity, which weakened the inter-splat bonding. As the spray angle decreased to 60°, the porosity reduced due to improved splat bonding resulting from effective tamping and hammering, despite the higher tangential velocity. At a 45° spray angle, the porosity increased again due to poor inter-splat bonding caused by the high tangential impact velocity.
- Fracture Toughness and Microstructure: Micro-scratch testing effectively evaluated the fracture toughness of the SS316L coatings. The results showed a strong correlation with the microstructure of the coating, highlighting that fracture toughness is influenced by both deformation ability and cohesion strength. The assessment employed a linear elastic fracture mechanics approach, with and without considering transition depth.

In conclusion, the spray angle plays a crucial role in determining the microstructure and inter-splat bonding of cold-sprayed SS316L coatings. A 90° spray angle offers superior performance in terms of scratch resistance, interfacial structure, and overall quality. Scratch testing has proven to be a valuable method for optimizing cold spray process parameters.

## Figures and Tables

**Table 1:** Process parameters used for the development of cold-sprayed SS316L coatings on SS304 substrates.

Sample Number	Gas Pressure (bar)	Gas Temperature (°C)	Spray Angle (°)	Feed Rate (rpm)	Stand-Off Distance (mm)
1	45	900	45	3	30
2	45	900	60	3	30
3	45	900	75	3	30
4	45	900	90	3	30

**Table 2:** Coating thickness at different spray angles after three passes.

Spray Angle	45°	60°	75°	90°
Coating thickness	200 ± 26 µm	494 ± 35 µm	735 ± 19 µm	747 ± 34 µm

**Table 3:** Scratch width to indenter diameter and applied load to scratch width ratio at each spray angle for the investigated coatings.

45°		60°		75°		90°	
Scratch Width-to-Indenter Diameter (W/D)	Applied Load-to-Scratch Width (P/W)	Scratch Width-to-Indenter Diameter (W/D)	Applied Load-to-Scratch Width (P/W)	Scratch Width-to-Indenter Diameter (W/D)	Applied Load-to-Scratch Width (P/W)	Scratch Width-to-Indenter Diameter (W/D)	Applied Load-to-Scratch Width (P/W)
0.18	0.27	0.16	0.30	0.17	0.3	0.18	0.28
0.26	0.38	0.24	0.41	0.24	0.41	0.26	0.39
0.31	0.48	0.30	0.49	0.30	0.50	0.31	0.49
0.35	0.56	0.34	0.59	0.33	0.61	0.37	0.54
0.38	0.65	0.37	0.66	0.39	0.65	0.40	0.62

## Data Availability

The original contributions presented in the study are included in the article; further inquiries can be directed to the corresponding author.
